# Distribution and Ecology of Cyanobacteria in the Rocky Littoral of an English Lake District Water Body, Devoke Water

**DOI:** 10.3390/life4041026

**Published:** 2014-12-16

**Authors:** Allan Pentecost

**Affiliations:** Freshwater Biological Association, Ferry Landing, Ambleside, Cumbria LA22 0LP, UK; E-Mail: Allan.pentecost@kcl.ac.uk; Tel.: +44-15394-42468

**Keywords:** mountain, ecology, littoral, distribution, exposure, lake

## Abstract

Cyanobacteria were sampled along two vertical and two horizontal transects in the littoral of Devoke Water, English Lake District. Profiles of cyanobacterium diversity and abundance showed that both attained a maximum close to the water line, but declined rapidly 20–40 cm above it. The distribution of individual species with height together with species and site ordinations showed that several taxa occurred in well-defined zones. A narrow “black zone” in the supralittoral was colonised mainly by species of *Calothrix*, *Dichothrix* and *Gloeocapsa* with pigmented sheaths. There was no evidence of lateral variation of species around the lake, but the height of the black zone correlated positively with wind exposure. The flora of Devoke Water is that of a base-poor mountain lake with some elements of a lowland, more alkaline water-body.

## 1. Introduction

The rocky littoral of British lakes is often colonised by cyanobacteria where they are sometimes revealed as a dark zone extending a short way above the mean water level. While the occurrence of these algae in this habitat has long been recognised there have been few detailed studies of the composition and distribution of the organisms responsible since the work of Godward on Windermere in the English Lake District [1]. Further afield, more recent studies have indicated that cyanobacteria colonising this habitat are often related to the water level, suggesting a relationship with the frequency of wetting and drying events and tolerance to desiccation. Since water absorbs the solar radiation there is a negative correlation between water depth and this variable. At, or above water level, both visible and ultraviolet radiation may be intense, particularly at high altitudes and several studies have addressed this issue in the lake littoral [2,3]. There is also evidence to suggest the chemical composition of the water [4], the nature of the substratum [5] and the prevailing climate [6] influence the flora.

This paper provides information on the littoral cyanobacterium flora of Devoke Water, a small rocky lake in the English Lake District. This lake was selected for its extensive rocky shores and exposed position. With prevailing westerly winds, the eastern shore of the lake is most exposed to wave action providing a range of exposures on the different shores. The aims of the investigation are three-fold; to examine the cyanobacterium flora and compare it with the littoral of other Cumbrian and continental lakes; investigate zonation patterns species associations and diversity above and below the water line and finally to determine the effect of exposure on the distribution and abundance of the cyanobacteria.

## 2. Experimental Section

**Figure 1:**
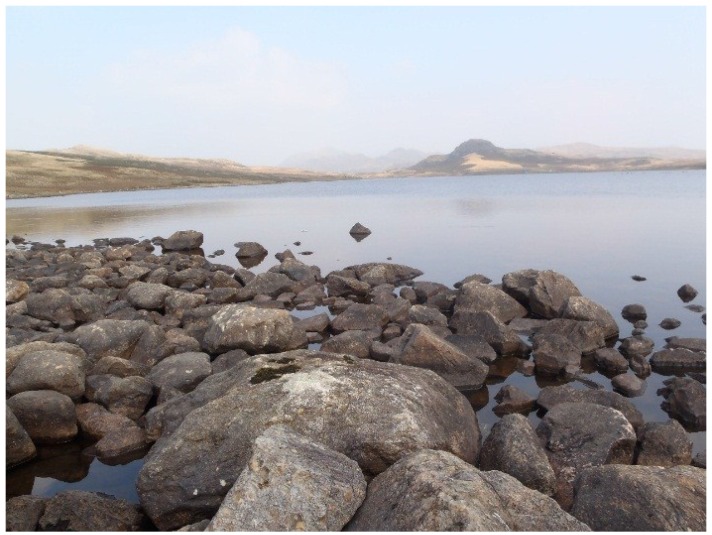
View of Devoke Water looking east from Station 1 showing littoral rocks and boulders colonised by cyanobacteria.

**Figure 2:**
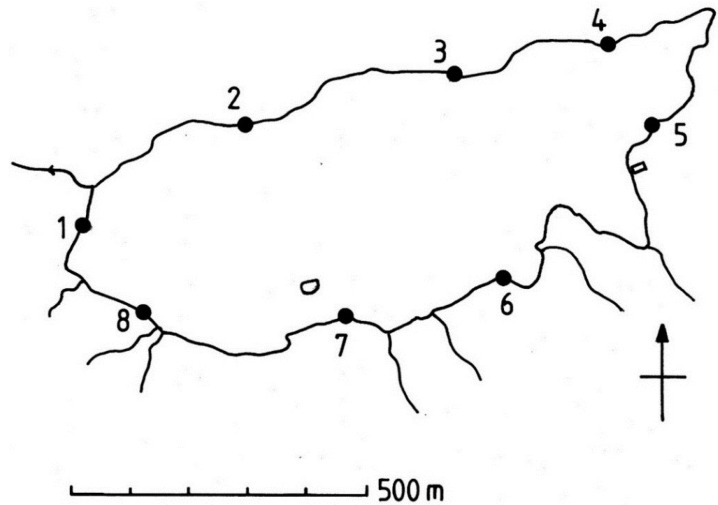
Map of Devoke Water showing the eight sampling stations.

Devoke Water is a small shallow lake 7 km east of the town of Ravenglass in Cumbria (Figure 1). The morphometry, palaeolimnology and general biology of this lake has been previously investigated and a range of limnological data are provided in Table 1. The catchment bedrock consists of granite and andesitic volcanic rocks overlain by glacial till and thin acidic peat soils [7]. Precipitation at this location is high, approximately 2200 mm per annum and is distributed throughout the year with small maxima in winter and late summer. Mean air temperature is close to 8.5 °C. Samples of cyanobacteria were collected from eight stations during September 2014 (Figure 2). Stations were situated at regular intervals around the shore and samples were removed with a sharp knife at water level and 10 cm above this level. In addition, more intensive sampling was undertaken at two of these stations: At 2 cm vertical intervals between +40 cm and −20 cm from the waterline, and at 5 cm intervals up to +100 cm and −50 cm. Location of sampling points was aided by a tape and level. Below the waterline, the scrapings were removed with the aid of a plastic pipette and the suspension transferred into a plastic vial. Adjacent samples of bryophytes and lichens were also removed for identification in the laboratory. At the time of sampling, the lake water level was 20 cm deep at the outflow.

**Table 1:** Limnological data for Devoke Water [8].

Factor	Value
Lat/long	54°21'N, 3°18'W
National grid reference	34/158969
Altitude	244 m
Surface area	35 ha
Maximum depth	16 m
pH	6.8
Alkalinity	66 μeq/L

Subsamples of algae representing a surface area of approximately 20 mm^2^ were examined under a light microscope at 500× magnification. Relative abundance was recorded on a five-point scale [9]: 1 single observation of species; 2 occasional, up to 5%; 3 frequent 5%–25%; 4 common 25%–50%; 5 dominant, >50%. All cyanobacteria were identified to species where possible with nomenclature following Whitton [10]. A few taxa required combination into larger groups owing to identification difficulties. *Calothrix parietina* and *Dichothrix orsiniana* are morphologically similar and distinguished mainly upon their degree of aggregation. Some intermediate forms were encountered and for some purposes, the two cyanobacteria were combined as *Calothrix/Dichothrix*. A similar problem was encountered with the species of *Stigonema* and of *Lyngbya* which were again combined into aggregate taxa. Associated eukaryotic algae, mostly chlorophytes and diatoms were with few exceptions, identified as growth-form only. At each station, the height of visible colonisation by cyanobacteria, revealed as a dark stain on the littoral rocks, was also recorded.

Frequency data were analysed using detrended correspondence analysis from the Canoco 4 statistical package [11]. Species that occurred less than 3 times in the study were removed from the analysis.

## 3. Results and Discussion

### 3.1. Results

#### 3.1.1. General Flora

At all eight stations, the littoral zone was dominated by cyanobacteria from water level up to +30 cm. Above 30 cm, lichens and bryophytes became significant while below the waterline, green algae and diatoms were at least as abundant as the cyanobacteria down to −50 cm.

Thirty one species of cyanobacteria were identified in the littoral zone (Table 2). The five most frequently encountered, in descending order were: *Lyngbya lagerheimii* s.l., *Gloeocapsa rupicola*, *Calothrix parietina*, *Gloeocapsa compacta* and *Dichothrix orsiniana*. These were closely followed by *Gloeocapsa kuetzingiana*, *Aphanocapsa fonticola* and *Stigonema mamillosum*. The frequencies of all species are presented in Table 2.

**Table 2:** Cyanobacteria recorded from the littoral of Devoke Water. Relationship with water level in cm shown where taxa are not figured in text.

Order/Genus	Species/Variety	Position Range in Relation to Water Level	Frequency of Occurrence
***Chroococcales***			
*Aphanocapsa*	*fonticola*		31
*A.*	*grevillei*	−40, +32	8
*Aphanothece*	*elabens*	−40, −2	10
*A.*	*microscopica*	−10, −20	2
*Chroococcus*	*minutus*	−12	1
*C.*	*pallidus*	−12, −50	5
*C.*	*turgidus*	−2, −25	2
*Gloeocapsa*	*compacta*		46
*G.*	*granosa*	−30	1
*G.*	*kuetzingiana*		32
*G.*	*punctata*		18
*G.*	*rupicola*		52
*Hydrococcus*	*rivularis*	−10, +10	3
*Merismopedia*	*punctata*	−30, +18	12
***Oscillatoriales***			
*Ammatoidea*	*normanii*	0, +10	3
*Homoeothrix*	*fusca*	−4, +32	16
*Lyngbya*	*lagerheimii s.l.*		71
*Microcoleus*	*lacustris*	0, +32	3
*Schizothrix*	*calcicola*	−12, 0	2
*S.*	*fuscescens*	0, +28	9
*S.*	*heufleri*	−6, +12	2
***Nostocales***			
*Calothrix*	*braunii*	−8, −16	1
*C.*	*epiphytica*	−10	1
*C.*	*parietina*		47
*Dichothrix*	*gypsophila*	+22	1
*D.*	*orsiniana*		33
*Nostoc*	*sp.*	−16	1
*Scytonema*	*mirabile*	0, +30	7
*S.*	*myochrous*	+10, +16	2
***Stigonematales***			
*Stigonema*	*mamillosum*		31
*S.*	*ocellatum*		15

#### 3.1.2. Vertical Zonation

**Figure 3:**
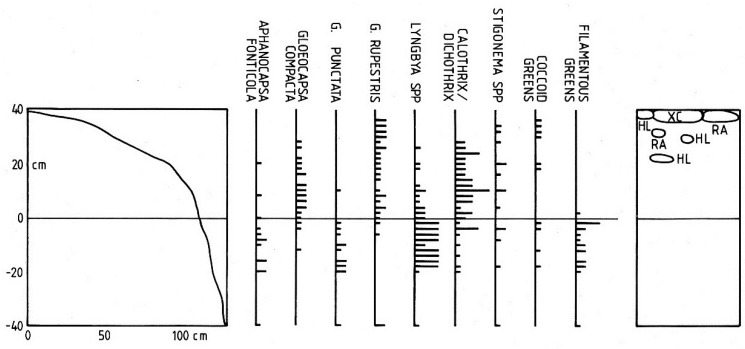
Distribution of the main littoral cyanobacteria at Station 1. Left, profile of lake edge; centre, relative abundance of cyanobacteria and green algae; right, distribution of associated bryophytes and lichens. *Hymenelia lacustris* (HL), *Racomitrium aciculare* (RA), *Xanthoparmelia conspersa* (XC).

**Figure 4:**
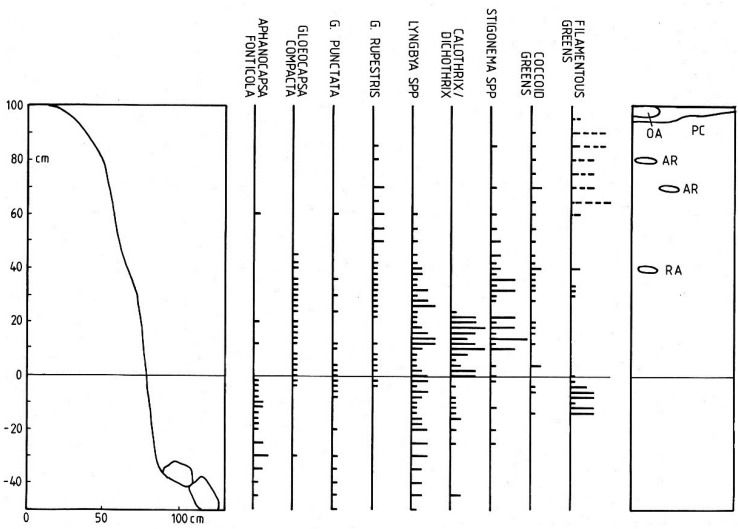
Distribution of main littoral cyanobacteria at Station 5. Explanation as in Figure 3. Additional bryophytes and lichens: *Andreaea rothii* (AR), *Ochrolechia androgyna* (OA), *Pertusaria corallina* (PC) and *Racomitrium aciculare* (RA).

Distributions of the most frequently encountered cyanobacteria, together with coccoid and filamentous green algae are shown in Figure 3 and Figure 4. At Station 1 (Figure 3) cyanobacteria extended up to 35 cm above water level, and several taxa were also encountered below the water. The cyanobacteria could be divided into three ecological groups: (a) those occurring predominantly above the waterline (*Gloeocapsa compacta*, *G. rupestris* and *Calothrix/Dichothrix*); (b) those found mainly below the waterline (*Gloeocapsa punctata*, *Lyngbya lagerheimii* s.l. and filamentous green algae) while the remaining group (c), appeared indifferent to water level and included *Aphanocapsa fonticola*, *Stigonema* spp and coccoid green algae.

At Station 5, the more exposed location at the eastern end of the lake (Figure 4) a similar pattern emerged although among the above-water level species, *Calothrix/Dichothrix* extended below the water level in small numbers while *Gloeocapsa punctata*, *Lyngbya lagerheimii* s.l. and *Stigonema* spp. were more widely distributed above water level. This transect also passed through an upper zone of the filamentous green alga *Klebsormidium crenulatum*. Overall, the transect revealed a similar assemblage of cyanobacterium species but with several of the common taxa extending further above the waterline. Where *Calothrix parietina* could be clearly distinguished from *Dichothrix orsiniana* in the *Calothrix/Dichothrix* pair, their vertical distribution was found to be almost identical. In addition, the two taxa in the “*Stigonema* spp.” group, *S. mamillosum* and *S. ocellatum* showed a similar range and zonation pattern. At both stations, several bryophytes and lichens shared the littoral zone, *Racomitrium aciculare* and *Hymenelia lacustris* being the most frequent (Figure 3 and Figure 4).

Of the remaining cyanobacteria, the Chroococcales *Gloeocapsa kuetzingiana* and *Merismopedia punctata* were frequently encountered, the latter being almost entirely below the waterline (Table 2). In the Oscillatoriales, several brown-sheathed taxa were encountered; *Homoeothrix fusca* was the most frequent, occurring above water and occasionally with the superficially similar *Ammatoidea normanii* and *Schizothrix fuscescens*.

**Figure 5:**
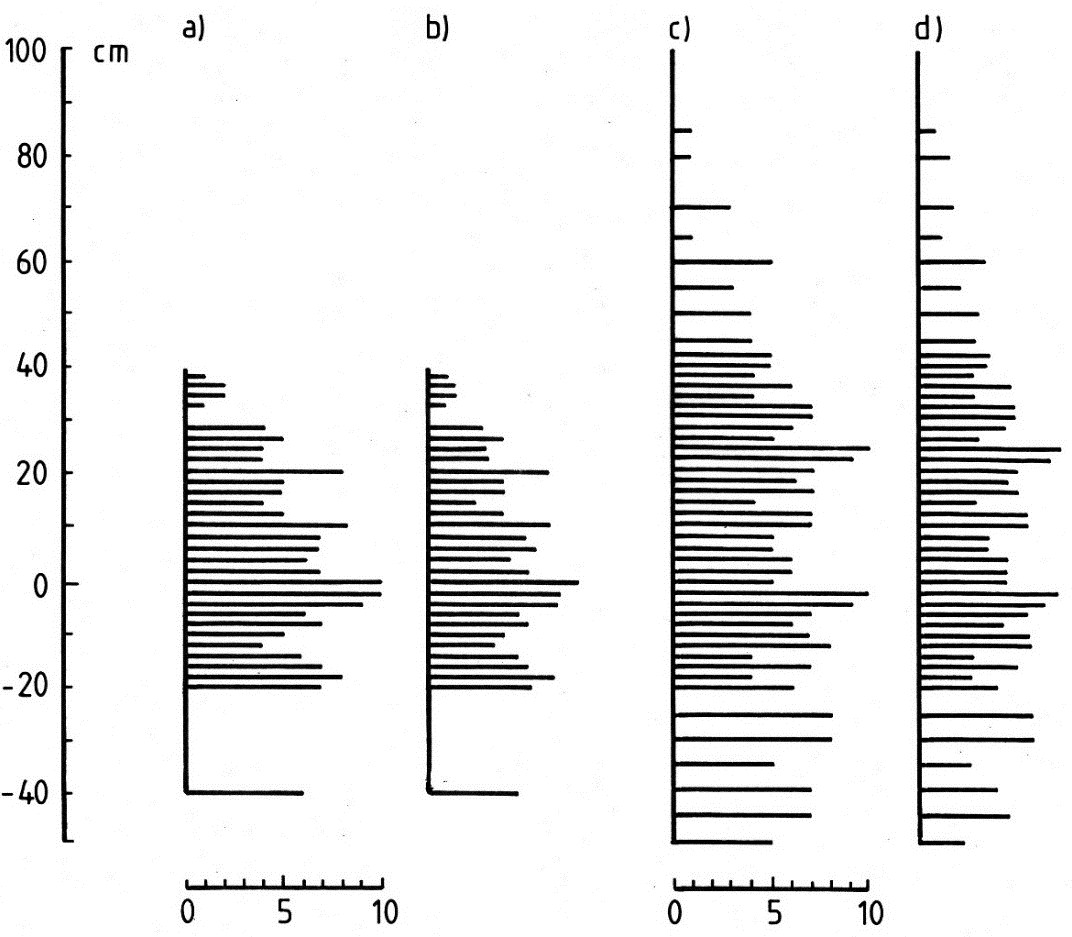
Overall diversity and abundance of littoral cyanobacteria in Devoke Water. (**a**) Species richness at Station 1; (**b**) species relative abundance at Station 1; (**c**) species richness at Station 5; (**d**) species relative abundance at Station 5.

Data obtained from the two principal sampling stations, 1 and 5 were used to provide profiles of diversity as species richness and relative abundance (Figure 5). Maximum species richness per sample (*n* = 10) occurred in the range −2 to +24 cm at both stations, and above 24 cm, it declined (Figure 5a,c). Below water level, richness remained high (average 6.9) down to 50 cm depth. Species relative abundance reflected closely the species richness at both stations (Figure 5b,d).

#### 3.1.3. Cyanobacterium Communities

**Figure 6:**
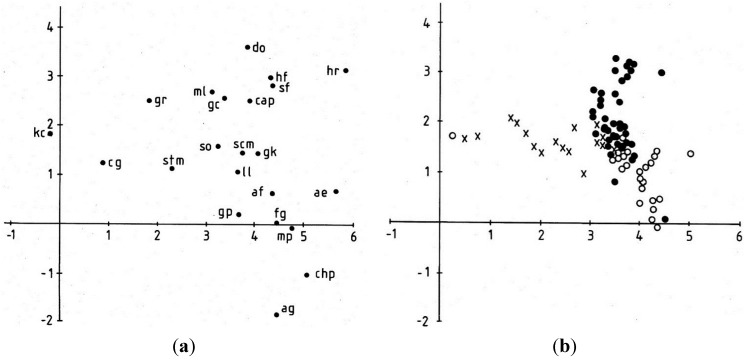
(**a**) Biplot of cyanobacterium species: af *Aphanocapsa fonticola*, ag *Aphanocapsa grevillei*, ae *Aphanothece elachista*, chp *Chroococcus pallidus*, cg coccoid green algae, fg filamentous green algae, gl *Gloeocapsa compacta*, gk *G. kuetzingiana*, gp *G. punctata*, gr *G. rupestris*, hr *Hydrococcus rivularis*, kc *Klebsormidium crenulatum* [green alga], mp *Merismopedia punctata*, an *Ammatoidea normanii*, hf *Homoeothrix fusca,* ll *Lyngbya lagerheimii* s.l., mL *Microcoleus lacustris*, sf *Schizothrix fuscescens*, cap *Calothrix parietina*, do *Dichothrix orsiniana*, scm *Scytonema mirabile*, stm *Stigonema mamillosum*, so *Stigonema ocellatum*; (**b**) Biplot of the 90 samples from the eight stations. Open circles represent samples taken below lake level, closed circles sites 0–30 cm above lake level. Crosses represent samples taken >30 cm above lake level.

A species ordination using detrended correspondence analysis is shown in Figure 6a. Eigenvalues for the first three axes of the ordination were 0.692, 0.304 and 0.172 representing 31.4% of the total variance. The scatterplot shows the positions of all cyanobacteria with frequencies of three or more in the total dataset (Table 2). A few taxa appear to be closely associated, namely *Homoeothrix fusca* (hf) with *Schizothrix fuscescens* (sf); *Microcoleus lacustris* (ml) with *Gloeocapsa compacta* (gc) and “filamentous green algae” (exluding *Klebsormidium crenulatum*) with *Merismopedia punctata* (mp). Outliers include *Klebsormidium crenulatum* (kc), *Ammatoidea normanii* (an) and *Aphanocapsa grevillei* (ag). Overall, there is a tendency for species preferring more xeric conditions to occur in the lower left of the ordination and those of more mesic/aquatic conditions in the upper right.

Sites were classified into three groups for the ordination shown in Figure 6b, namely those between 0 and 30 cm above water level, those >30 cm and those between 0 and −50 cm. A fairly good separation of these groups is apparent, showing again a gradient of increased wetness from left to right, with a dense cluster of sites in the middle-right of the scatter.

#### 3.1.4. Lateral Variation

**Figure 7:**
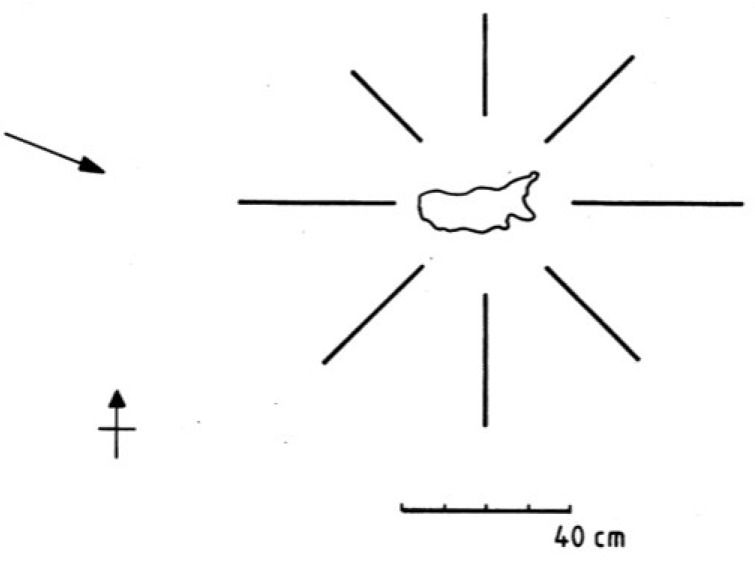
Height of visible cyanobacterium colonisation above the lake level at the eight sampling stations. Arrow shows prevailing wind direction.

**Figure 8:**
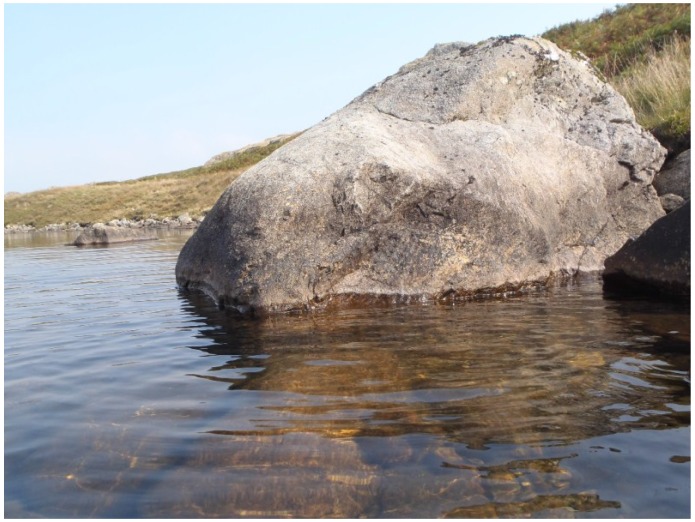
Littoral zone of Devoke Water close to Station 5 showing a dark line on the boulder representing the limit of cyanobacterial colonisation at approximately 40 cm above water level.

In the site ordination (Figure 6b) the eight stations where cyanobacteria were sampled at 0 cm and 10 cm showed no systematic variation in relation to wind exposure suggesting that it did not influence the flora unduly. The height of cyanobacterium colonisation above water level at these stations is depicted in Figure 7. The level ranged from 23 to 40 cm above lake level and highest at Station 5 which was most exposed to the prevailing westerly winds (Figure 8).

### 3.2. Discussion

This study demonstrates a zonation of epilithic cyanobacteria in the littoral zone of Devoke Water, with a rapid decline in species diversity and abundance with height above water level. Species richness overall compares favourably with other studies, Kann [4] for example, reported 30 taxa from the littoral of Lake Erken, Sweden. In Devoke Water, two coccoid cyanobacteria with pigmented sheaths were common in a well-defined “black zone” extending up to 40 cm above water level. These were *Gloeocapsa compacta* and *G. rupestris*, both widely distributed subaerial species. Red-sheathed *G. rupestris* is more frequently associated with low-pH waters. Their co-occurrence at Devoke Water where water pH is close to neutrality is not surprising since their low alkalinity makes the water weakly buffered against pH changes. The red sheath pigment gloecapsin, present in these species turns blue in alkaline water and some authors combine the red- and blue-sheathed species into a single taxonomic unit [4,12]. *Gloeocapsas* with pigmented sheaths have been widely reported from the black zone of lakes, such as the oligotrophic Zellersee [13] and calcareous Lake Ohrid [14]. The preponderance of pigmented sheathed cyanobacteria above the water line is probably a response to reduced growth rate combined with high levels of ultraviolet radiation.

Two filamentous members of the Nostocales, *Calothrix parietina* and *Dichothrix orsiniana* were also common in the black zone. The former is a well-known subaerial species [15] and is widespread in the littoral of other lakes such as Lago di Tovel [16], and Windermere [1]. The related *Dichothrix orsiniana* has been reported from Lake Erken and the Grössen Ploner See littoral [12] and was once abundant in parts of Windermere [1]. Several other filamentous cyanobacteria were found in this zone. *Microcoleus lacustris* was uncommon but is an occasional member of this zone in other English Lake District waters such as Derwent Water (unpublished data). The remaining filamentous species were all brown-sheathed forms and of these, *Ammatoidea normanii* was the least common. *Homoeothrix fusca* and *Schizothrix fuscescens* were more common and closely associated with one another. The former has been reported as abundant in Windermere [1], the Titisee [17] and the Zellersee [13]. The latter is physiographically and chemically similar to Devoke Water. *Schizothrix fuscescens* is sometimes found forming mats in mountain rock seepages in England and its occurrence in the littoral of lakes is to be expected.

Several cyanobacteria failed to show strong vertical zonation patterns and occurred with approximately equal frequency above and below the water level. *Lyngbya lagerheimii* s.l. was the most abundant of these, forming a thin irregular stratum on the rock surface. This species has narrow trichomes (2–3 μm wide) within a well-defined colourless sheath, but the cells were of variable length and in some trichomes they possessed terminal polyphosphate granules. Several closely related species may have been included in this morphotype. *Lyngbya* is rarely reported epilithic from the lake littoral but species of the closely related genus *Phormidium* are widespread [1,4,5,14,18]. The two species of *Stigonema* showed only a weak zonation. In Britain, *Stigonema* occurs frequently in the rocky littoral of lakes of low alkalinity such as Scoat Tarn, Cumbria (author unpublished) although there are few reports in the literature. They appear to be more conspicuous in alpine or subalpine lakes [6].

A number of cyanobacteria, widely reported from the littoral of continental lakes appear to be absent from the rocky littoral of Devoke Water. These include species of *Chamaesiphon*, *Tolypothrix* and *Chlorogloea microcystoides*. *Chamaesiphon* is a common epiphyte in many British lakes and its apparent absence from Devoke is unexplained, although it does occur as an epiphyte there. *Scytonema*, a close relative of *Tolypothrix*, did occur in the black zone of Devoke Water but it was not common and these genera were not reported from the chemically similar Zellersee [13]. The cyanobacterium flora of the Devoke littoral combines elements of a base-poor high-latitude lake and a base-rich lowland lake owing to its geographical position and catchment geology.

The zonation of littoral cyanobacteria was been recognised in many rocky lakes [1,12,19] and the dark supralittoral region named the “schwarzbandzone” [12]. In Lake Ohrid, it was designated the “*Gloeocapsa-Scytonema*” zone [14] though it is clear that species composition varies from lake to lake. At Devoke Water, the zone extends around the lake and was higher at the eastern, more exposed end. In addition, several taxa extended further above the water line at this location, demonstrating the importance of wave action, water splash and spray. However the influence of lake water is limited in extent, since terrestrial algae (e.g., *Klebsormidium crenulatum*), bryophytes and lichens colonised and out-competed the cyanobacteria c. 50 cm above the water level. Black bands have been reported up to similar heights in a number of other lakes [4,12,18,20]. The relationship between fetch, wave height and lake size indicates that band height should be positively correlated to these factors but there is as yet no evidence to support this. The lateral variation at Devoke Water did not reveal any outstanding differences in the cyanobacterium flora and there is no evidence to indicate a systematic difference in the species composition along different parts of the shore. The lake was surrounded by gently sloping ground, but in more rugged terrain, where strong shading from steep cliffs could occur, differences caused by aspect may be expected.

One of the challenging aspects of lake littoral ecology is the assessment of water level. This is important as it allows the supralittoral and sublittoral communities to be clearly defined. Identification of maximum, minimum and mean water level is important but impractical for remote, little visited lakes such as Devoke Water. An alternative is to establish a datum related to the water level at the lake outflow. This method was adopted here and permits any future work at the site to be measured against a known lake level. The datum used at Devoke Water would have been several centimeters below the mean water level as it was taken after three weeks of dry weather.
